# Multiplex bead analysis of vitreous and serum concentrations of inflammatory and proangiogenic factors in diabetic patients

**Published:** 2008-03-27

**Authors:** Richard Maier, Martin Weger, Eva-Maria Haller-Schober, Yosuf El-Shabrawi, Andreas Wedrich, Anna Theisl, Reingard Aigner, Alfred Barth, Anton Haas

**Affiliations:** 1 Department of Ophthalmology, Medical University of Graz, Graz, Austria; 2 Division of Nuclear Medicine, Medical University of Graz, Graz, Austria; 3 Institute of Management Science, Division Ergonomics and Organisation, Vienna University of Technology, Vienna Austria

## Abstract

**Purpose:**

To investigate the role of inflammatory and angiogenic factors in the pathogenesis of diabetic retinopathy, we determined, in diabetic patients and controls, vitreous and serum concentrations of interferon-induced protein (IP)-10, monocyte chemoattractant protein (MCP)-1, macrophage inflammatory protein (MIP)-1α, MIP-1β, regulated upon activation, normal T-expressed and secreted (RANTES), and vascular endothelial growth factor (VEGF).

**Methods:**

We recruited 36 probands with type 2 diabetes mellitus (15 noninsulin-dependent and 21 insulin-dependent) and 69 normal controls. Using Cytometric Bead Array Technology, we measured vitreous and serum concentrations of IP-10, MCP-1, MIP-1α, MIP-1β, RANTES, and VEGF.

**Results:**

In diabetic patients the mean vitreous levels of IP-10, MCP-1 and VEGF were significantly higher compared normal controls. [IP-10 (pg/mL) 254.84 +/−311.67 versus 78.90 +/− 67.94 (p<0.001); MCP-1 (pg/mL) 1127.14 +/− 738.91 versus 700.80 +/−419.21 (p=0.002); VEGF (pg/mL) 954.98 +/− 2315.09 versus 37.90 +/− 28.51(p<0.001)]. Vitreous levels of VEGF correlated with vitreous levels of both IP-10 and MCP-1 (p<0.05). MIP-1β, RANTES, and VEGF mean serum levels were significantly raised in diabetic probands while IP-10, MCP-1, and MIP-1α serum levels showed no significant elevation compared to controls [IP-10 (pg/mL) 346.20 +/− 287.36 versus 328.74 +/−352.35 (p=0.88); MCP-1(pg/mL) 133.10 +/− 89.10 versus 141.47 +/− 222.15 (p=0.50); MIP-1β (pg/mL) 184.40 +/− 100.20 versus 139.56 +/− 151.38 (p=0.003); RANTES (pg/mL) 51336.23 +/− 19940.31 versus 33629.2 +/− 33301.0 (p=0.002); VEGF (pg/mL) 304.88 +/− 257.52 versus 154.45 +/− 114.78 (p<0.001)].

**Conclusions:**

Our results suggest that in diabetics, there is an upregulation of IP-10, MCP-1, and VEGF in the vitreous and an upregulation of MIP-1β, RANTES, and VEGF in the serum. These findings support the concept of an angiogenic and inflammatory element in the development of diabetic retinopathy.

## Introduction

Diabetic retinopathy (DR) is the most common cause of visual loss in the working population and one of the primary reasons for blindness worldwide [1,2]. Diabetic vessel changes may induce ischemia, leading to an upregulation of angiogenic factors in the retina. Aside from the angiogenic component, there is a large body of evidence indicating that inflammation is an important event in the pathogenesis of DR [3]. Leukocytes play a major role in the hypoxic retina by producing inflammatory cytokines. Evidence from the retinal ischemia-reperfusion injury model has demonstrated that inflammatory chemokines substantially contribute to inducing retinal damage by infiltration of ocular tissue by leukocytes [4].

A recent study investigated vitreous chemokine levels by multiplex bead analysis in 58 patients with vitreoretinal disorders such as uveitis, choroidal neovascularization, proliferative vitreoretinopathy, and proliferative DR (PDR) [5]. The present study is the first to examine interferon-induced protein (IP)-10, monocyte chemoattractant protein (MCP)-1, macrophage inflammatory protein MIP-1α, macrophage inflammatory protein MIP-1β regulated upon activation, normal T-expressed and secreted (RANTES), and vascular endothelial growth factor (VEGF) in the vitreous and serum by multiplex bead analysis in a large number of diabetic patients compared to controls.

Chemokines are small molecular weight proteins that guide the migration of responsive cells. Cells attracted by chemokines follow the direction of increasing chemokine concentration. In addition to chemoattraction, inflammatory chemokines cause activation of leukocytes. Chemokines are categorized into four subgroups: CXC, CC, C, and CX3X [6].

IP-10 is a CXC chemokine that contributes to the T-helper type 1immune reactivity. It is secreted by monocytes, endothelial cells, and fibroblasts, and it promotes chemoattraction for monocytes and T-cells [7]. Earlier studies described elevated serum IP-10 concentrations as a risk factor for type 1and type 2 diabetes mellitus, and it has been suggested that vitreous levels of IP-10 are increased in patients with PDR [8–10]. The present study is the first to determine IP-10 in the vitreous by Cytometric Bead Array (CBA) technique.

MCP-1, MIP-1α, MIP-1β, and RANTES are members of the CC chemokines. MCP-1 recruits immune cells, such as monocytes. It is produced by retinal endothelial cells and has been implicated in leukostasis in the hypoxic retina [3,4].

In animal models raised MCP-1 mRNA levels after retinal hypoxia induction were determined [11]. In rats increased expression of IP-10, MCP-1, MIP-1α, and MIP-1β was found in the hypoxic inner retina [4]. Previous data presumed MCP-1 as a potential factor in the proliferative phase of DR [12].

MIP-1α and MIP-1β are produced by macrophages and activate human granulocytes such as neutrophils, eosinophils, and basophils, which may lead to acute neutrophilic inflammation [13]. MIP-1α and MIP-1β activate granulocytes and induce the release of proinflammatory interleukins such as interleukin-1, interleukin-6, and tumor necrosis factor-α [14,15]. MIP-1α mediates the recruitment of monocytes. In a mouse model, MIP-1α has been identified as a potent inducer of retinal neovascularization during postischemic inflammation [16]. The retinal ischemia-reperfusion model showed an upregulation of MIP-1β in the retinal vessels [4]. We therefore conclude, that MIP-1β may be involved in the inflammatory element of pathogenesis of retinal neovascularization. An upregulation of serum levels of MIP-1α and MIP-1β has been found in type 1 diabetics [17].

RANTES, produced by inflammatory cells, retinal endothelial cells, and retinal pigment epithelial cells, attracts T-cells, eosinophils, and basophils. In addition, RANTES activates natural killer cells [18,19]. In tumor model systems, RANTES seems to be a potent angiogenic factor and may be involved in retinal neovascularization in diabetic patients [20,21]. Immunohistochemical investigations of a donor eye with DR showed the presence of RANTES in the diabetic retina compared with the retina of a nondiabetic subject [3]. Further, multiplex bead analysis detected RANTES in the vitreous of patients with PDR [5].

VEGF plays a major role in ocular neovascularization. VEGF is not only a major mediator of retinal angiogenesis, but also a potent inducer of vasopermeability [22]. The expression of VEGF is upregulated in the retinal pigment epithelium, glial cells, and vitreous fibroblasts in diabetic patients [23].

CBA was used to determine vitreous and serum levels of IP-10, MCP-1, MIP-1α, MIP-1β, RANTES, and VEGF. The technology has already been used for the measurement of cytokines and growth factors in vitreous, aqueous humor, plasma and serum samples as well as in tissue culture supernatants [24–28]. The determination of cytokines and growth factors in the vitreous by CBA promises to be of substantial benefit for ophthalmologic investigations because numerous parameters can be measured in parallel using a comparatively small sample volume. Furthermore CBA is faster and more cost effective than ELISA technology.

For an improved management of DR a better understanding of the pathogenesis is crucial. To elucidate the role of inflammatory and angiogenic factors in the development of DR we measured both vitreous and serum levels of IP-10, MCP-1, MIP-1α, MIP-1β, RANTES, and VEGF in diabetics and normal controls.

## Methods

### Study population

Diabetes was diagnosed according to the 1999 World Health Organization criteria [29]. For all probands, the indications for pars plana vitrectomy were diabetic macular edema, epiretinal membranes, and macular holes. In the diabetes group, 10 had no DR, 10 had non-PDR, and 16 had PDR. Exclusion criteria were previous intraocular surgery, earlier intravitreal therapies, photocoagulation in the preceding three months, uveitis, trauma, vitreous hemorrhage, and retinal detachment. Informed consent was obtained from all probands and controls before entering the study.. Research was performed in accordance with the ethical standards of the Declaration of Helsinki [30].

### Gender and age breakdown

Patients were recruited from the Department of Ophthalmology of the Medical University Graz. The population of this case-control study consisted of 36 patients with diabetes mellitus (21 non-insulin dependent, 15 insulin dependent) and 69 non-diabetic control subjects. The mean age of the 36 diabetic subjects (15 men and 21 women) was 66.2 years (SD+-12.2 years). Twenty-one probands were insulin-dependent and 15 probands were non-insulin-dependent. The mean age of the 69 non-diabetic control subjects (23 men and 46 women) was 67.9 years (SD+-10.3 years). With regard to age no significant difference between both groups was observed (p>0.05). The mean hemoglobin A1c (HbA_1_c) of the diabetic subjects was 7.40 +-1.11% compared to 5.7 +-0.2% in control subjects (normal range HbA1c: 4.0 - 6.0%) (p<0.05).

### Sample collection and preparation

#### Vitreous

Undiluted vitreous samples were obtained during a standard three-port pars plana vitrectomy. After the patient was anesthetized, vitreous fluid (approximately 1 mL) was collected via pars plana using a 20- or 23-gauge vitreous cutter on a 5 mL-syringe after the infusion port was made without running the fluid on. The specimen was immediately transferred to a sterile plastic tube on ice. Promptly the sample was centrifuged at 5000 rpm for 10 min, aliquoted, and stored at –70 °C until assayed.

#### Serum

Blood samples were obtained by venous puncture directly before vitrectomy, and immediately placed on ice. Directly each sample was centrifuged at 3000 rpm for 10 min, aliquoted, and stored at –20 °C until assayed. IP-10, MCP-1, MIP-1α, MIP-1β, RANTES, and VEGF were determined in serum as well as in vitreous samples by CBA.

### Cytometric bead array

Concentrations of six different angiogenic factors were determined in both serum and vitreous samples using the BD^TM^ CBA Flex Set System and BD^TM^ Human Soluble Protein Master Buffer Kit (CatNo. 558246, BD Bioscience-PharMingen, San Diego, CA) and the BD^TM^ CBA Flex Sets for the measurement of IP-10 (CatNo. 558280, BD Bioscience-PharMingen), MCP-1 (CatNo. 558287, BD Bioscience-PharMingen), MIP-1α (CatNo 558325, BD Bioscience-PharMingen), MIP-1β (CatNo 558288, BD Bioscience-PharMingen), RANTES (CatNo 558324, BD Bioscience-PharMingen), and VEGF (CatNo. 558336, BD Bioscience-PharMingen) levels. Each BD^TM^ CBA Flex Set contained one bead population with distinct fluorescence intensity as well as the appropriate phycoerythrin (PE) detection reagent and standard. Five bead populations coated with capture antibodies specific to IP-10, MCP-1, MIP-1α, MIP-1β, and VEGF were mixed with each other. The CBA for RANTES was run separately because the serum samples had to be prediluted. RANTES was also determined in serum and vitreous samples by ELISA technique (Quantikine RANTES ELISA, R&D Systems, Minneapolis, MN). The tests were performed according to the manufacturer’s advice, and samples were run in duplicate.

As previously described [24] the mixed bead populations as well as the single bead population for RANTES were incubated with recombinant standards or test samples to form sandwich complexes. After addition of PE-conjugated detection antibodies, the samples were incubated again and then resolved in the FL-3 channel of a FACSCalibur flow cytometer (BD Bioscience). The results were generated in graphic and tabular format by using the CBA analysis software (BD Bioscience-PharMingen). The assay sensitivities were as follows: IP-10 0.4 pg/mL, MCP-1 1.3 pg/mL, MIP-1α 4.6 pg/mL, MIP-1β 1.4 pg/mL, RANTES 3.2 pg/mL, and VEGF 39.2 pg/mL.

For the detection of IP-10, MCP-1, MIP-1α, MIP-1β, and VEGF, serum and vitreous samples were run without predilution. For the detection of RANTES, serum samples were prediluted 1:50, and the vitreous samples were tested undiluted. The tests were performed and analyzed according to the manufacturer’s advice. In brief, 50 µL of the five mixed as well as the single capture beads were mixed with 50 µL of the provided standards or samples (serum, vitreous) and incubated in the dark for 1 h at room temperature. Subsequently 50 µL of the mixed as well as the single PE detection reagent(s) were added, and the samples were incubated in the dark for 2 h. After the second incubation step, the samples were washed, centrifuged (at 200xg for 5 min) and resuspended in 300 µL of wash buffer as previously described [24]. The BD FACSCalibur flow cytometer was calibrated with setup beads and 300 events were acquired for each factor and each sample, respectively. Individual analyte concentrations were indicated by their fluorescence intensities (FL-2) and were computed by using the respective standard reference curve and BD CBA software.

### Statistical analysis

Data were analyzed using the statistical package for social sciences (SPSS Version 11.0) for Windows. Group differences between samples from diabetics and controls were analyzed using a Student two-tailed *t*-test or two-tailed Mann–Whitney test depending on normality assumptions and homogeneity of variances. All tests were performed at an error level of 5%. Due to multiple univariate testing, the Bonferroni correction algorithm of error I level was applied to retain the global error level at 5%.

## Results

The normal range for HbA_1_c is 4.0 – 6.0%. After adjusting for age, sex, HbA_1_c, and C-reactive protein, we observed significantly higher levels of interferon-induced protein (IP)-10, monocyte chemoattractant protein (MCP)-1, and vascular endothelial growth factor (VEGF) in vitreous samples of diabetics compared to those in controls (Table 1).

**Table 1:** IP-10, MCP-1, MIP-1α, MIP-1β, RANTES, and VEGF in the vitreous samples of diabetic and control participants (pg/mL)

	Diabetics	Controls	
	Mean +/− SD (Range)	Mean +/− SD (Range)	p-value
IP-10	254.84 (311.67)	78.90 (67.94)	<0.001*
	10.00–1290.30	10.00–289.33	
MCP-1	1127.14 (738.91)	700.80 (419.21)	0.002*
	244.27–3330.50	86.97–2177.01	
MIP-1α	**	**	-
MIP-1β	**	**	-
RANTES	**	**	-
VEGF	954.98 (2315.09)	37.90 (28.51)	<0.001*
	10.00–13258.40	10.00–142.63	

Interferon-induced protein (IP)-10, monocyte chemoattractant protein (MCP)-1, macrophage inflammatory protein (MIP)-1α, macrophage inflammatory protein (MIP)-1β, RANTES (regulated upon activation, normal T-expressed and secreted) and vascular endothelial growth factor (VEGF) levels in the vitreous of diabetics and control samples given in picograms per milliliter. The single asterisk (*) indicates significant after Bonferroni correction p<0.006 (α=0.05) and the double asterisk (**) indicates, that the factor was below the detection limit. SD means standard deviation.

Mean serum levels of MIP-1β, RANTES, and VEGF were significantly raised in the diabetic group compared to controls. Mean IP-10 and MCP-1 serum levels demonstrated no significant elevation in diabetic probands compared to controls (Table 2). In both vitreous and serum, MIP-1α levels were below detection limits. Vitreous VEGF levels were positively correlated with vitreous IP-10 and MCP-1 levels (p=0.001). HbA1c levels in diabetic individuals were positively correlated with vitreous levels of IP-10, MCP-1, and VEGF (p<0.05). Only serum levels of MCP-1 were found to be significantly elevated among insulin dependent diabetic patients compared to controls, but not among non-insulin dependent diabetic patients (164.02 pg/mL ± 85.57 versus 141.47 pg/mL ± 222.15) (p<0.05)). .Vitreous and serum levels of all investigated parameters did not correlate with the stage of DR (p>0.0.5).

**Table 2:** IP-10, MCP-1, MIP-1α, MIP-1β, RANTES, and VEGF in serum samples from diabetic and control participants (pg/mL)

	Diabetics	Controls	s
	Mean +/− SD (Range)	Mean +/− SD (Range)	p-value
IP-10	346.20 (287.36)	328.74 (352.35)	0.88
	28.06–1586.90	56.06–2809.70	
MCP-1	133.10 (89.10)	141.47 (222.15)	0.5
	10.00–360.90	10.00–1850.34	
MIP-1 α	**	**	-
MIP-1β	184.40 (100.20)	139.56 (151.38)	0.003*
	43.07–403.61	9.21–1187.32	
RANTES	51336.23 (19940.31)	33629.25 (33301.01)	0.002*
	5800.00–99600.0	22.30–129100.0	
VEGF	304.88 (257.52)	154.45 (114.78)	<0.001*
	10.00–1196.40	10.00–563.34	

Interferon-induced protein (IP)-10, monocyte chemoattractant protein (MCP)-1, macrophage inflammatory protein (MIP)-1α, macrophage inflammatory protein (MIP)-1β, RANTES (regulated upon activation, normal T-expressed and secreted) and vascular endothelial growth factor (VEGF) levels in the serum of diabetics and control samples given in picograms per milliliter. The single asterisk (*) indicates significant after Bonferroni correction p<0.006 (α=0.05) and the double asterisk (**) indicates, that the factor was below the detection limit. SD means standard deviation.

## Discussion

The etiologic key components of DR are hypoxia-induced inflammation and angiogenesis. Previous studies investigated either vitreous or serum levels of angiogenic and inflammatory factors by multiplex bead analysis [4,5]. In the present case-control study both vitreous and serum concentrations of IP-10, MCP-1, MIP-1α, MIP-1 β, RANTES and VEGF were determined by multiplex bead analysis in 105 patients. After multivariable adjustment, mean levels of IP-10, MCP-1, and VEGF were found to be significantly increased in the vitreous of diabetics compared to controls. There was no significant correlation between vitreous and serum levels of diabetic probands, suggesting that IP-10, MCP-1, and VEGF levels in the vitreous reflected local production. HbA1c levels in diabetics were positively correlated with vitreous levels of IP-10, MCP-1, and VEGF. Mean serum levels of MIP-1β, RANTES, and VEGF showed significant elevation in diabetics compared to controls.

Increased levels of inflammatory and angiogenic factors in the vitreous of diabetic probands could be the result of three pathways. First, it is hypothesized that the breakdown of the blood-retina barrier leads to raised levels of inflammatory and angiogenic factors in the vitreous of diabetics [31,32]. A second possibility is the expression of inflammatory and angiogenic factors by cells within the vitreous fluid, such as macrophages, monocytes, glial cells, and retinal pigment epithelial cells [23]. Third, chemokines and angiogenic factors are expressed by the hypoxic retina as shown in the retinal ischemia-reperfusion model [4]. These different pathways might explain the differences between serum and vitreous levels of some of the factors.

This is the first study to determine, by multiplex bead analysis, IP-10 in the vitreous of diabetics . We observed raised IP-10 levels in the vitreous of diabetic patients, which is in accordance to the results of Abu-el-Asrar et al., who found raised IP-10 vitreous levels in patients with PDR compared to patients with retinal detachment [33]. It has been shown that IP-10 expression may be induced by VEGF in endothelial cells [34]. Our results support this concept as increased vitreous IP-10 levels were significantly positively correlated with increased VEGF levels, indicating that there may be a VEGF-induced release of IP-10 in the vitreous. The lack of association between VEGF and IP-10 in the serum is in accordance with previous findings [8,35].

MCP-1 is an angiogenic chemokine and may also be induced by VEGF [36]. It has been demonstrated that the angiogenic effect of MCP-1 is completely inhibited by a VEGF inhibitor [37]. A positive regulatory feedback loop between VEGF and MCP-1 expression by vascular endothelial cells has been suggested [33]. MCP-1 is a mediator of photoreceptor apoptosis in retinal disorders, such as DR [38]. Immunohistochemical examination of the human retina has revealed an augmented expression of MCP-1 by retinal endothelial cells [3]. In the present study significantly increased MCP-1 levels in the vitreous of diabetics were found. Raised MCP-1 levels in the vitreous indicate DR activity [39]. Additionally, a significant positive correlation between MCP-1 and VEGF in the vitreous could be shown. In accordance with our results, raised levels of MCP-1 in vitreous humor of patients with vitreoretinal disorders such as PDR were reported [33]. In insulin-dependent diabetics, our data showed significant elevation of mean serum MCP-1 levels compared to controls (data not shown). In noninsulin-dependent probands, serum MCP-1 levels were not raised compared to controls. This finding is likely due to our type 2 diabetics having a higher body mass index than our controls. Chemokines such as MCP-1 are released by adipocytes, and levels are body mass index correlated [35]. Therefore, the activity within the adipose tissue may lead to raised MCP-1 serum levels in type 2 diabetics.

In the vitreous, RANTES levels were below the detection limit in both diabetics and controls, as confirmed by ELISA technique. In contrast, Banerjee et al. used multiplex bead analysis and detected RANTES in the vitreous of patients with PDR [5]. Serum RANTES levels of our diabetic probands were significantly raised compared to controls. Our findings are supported by other studies using the ELISA technique (3,9).

MIP-1α mediates the recruitment of macrophages in several inflammatory diseases [10]. Vitreous and serum levels of MIP-1α and the vitreous level of MIP-1β were below detection limits in diabetic and control groups, which is in accordance with previous studies [3,33,40]. In contrast, Banerjee et al. detected MIP-1β and RANTES in the vitreous of patients with PDR [5]. In the study of Banerjee et al., vitreous hemorrhage was not an exclusion criterion and only diabetic patients with PDR were included, while in our study diabetics with and without DR were included and vitreous hemorrhages were excluded. Serum concentrations of MIP-1β were significantly raised in our diabetic study participants. Type 2 diabetes is associated with both T-helper cells type 1 (MIP-1β) and T-helper cells type 2 (RANTES)-associated chemokines [35].

VEGF plays an important role in retinal neovascularization in DR [41]. We determined significantly increased VEGF levels in vitreous and serum in diabetics compared to controls, which correlates with that seen in two other studies [42,43].

Our results support the concept of an inflammatory and angiogenic element in the development of DR because of significantly elevated levels of IP-10, MCP-1, and VEGF in the vitreous. Furthermore, we observed in diabetic patients a significant positive correlation between increased vitreous levels of IP-10, MCP-1, and VEGF. The cross-talk of these factors seems to be critical for the development of DR. The results show strong evidence for an angiogenic and inflammatory component in the development of DR. Additional studies are needed to provide better understanding of the interaction between inflammatory and angiogenic factors since new therapeutic approaches involve the neutralization of these factors in the vitreous of diabetic patients.
